# Effects of different seat inclination angles on lumbar dynamic response and injury during lunar-earth reentry

**DOI:** 10.3389/fbioe.2024.1395114

**Published:** 2024-06-11

**Authors:** Mengmeng Jin, Jiatao Wang, Qianxiang Zhou, Pan Guo, Jingfei Zhang, Yi Wang

**Affiliations:** ^1^ School of Mechanics and Safety Engineering, Zhengzhou University, Zhengzhou, China; ^2^ Key Laboratory for Biomechanics and Mechanobiology of the Ministry of Education, School of Biological Science and Medical Engineering, Beihang University, Beijing, China; ^3^ National Engineering Research Center for Advanced Polymer Processing Technology, Zhengzhou University, Zhengzhou, China; ^4^ Department of Physical Education, Renmin University of China, Beijing, China

**Keywords:** inclination angles, reentry load, lumbar spine injury, finite element modeling, biomechanics

## Abstract

The inclination angle of the spacecraft seat is related to the astronaut’s reentry angle, which in turn affects the safety of the astronauts. This study quantitatively analyzed the effects of different seat inclination angles on astronauts’ lumbar spine injuries using the finite element method during the Lunar-Earth reentry. Firstly, a finite element model of the astronaut’s lumbar spine was constructed based on reverse engineering technology, and the effectiveness of the model was verified through mesh sensitivity, vertebral range of motion, and spinal impact experiments. Then, simulation calculations were carried out for different seat inclination angles (0°, 10°, 20°, and 30°) under the typical reentry return loads of Chang’e 5T1 (CE-5T1) and Apollo 10, and the prediction and evaluation of lumbar spine injuries were conducted in conjunction with the biological tissue injury criteria. The results indicated that the stress on the vertebrae and annulus fibrosus increased under both reentry loads with the rise of the seat inclination angle, but the increasing rates decreased. When the acceleration peak of CE-5T1 approached 9G, the risk of tissue injury was higher under the seat angle exceeded 20°. According to the Multi-Axis Dynamic Response Criteria for spinal injury, neither of the two load conditions would directly cause injury to the astronauts’ lumbar spine when the seat inclination angle was below 30°. The study findings provide a numerical basis for designing and improving the spacecraft’s inclination angle in crewed lunar missions, ensuring the safety of astronauts.

## 1 Introduction

The 21st century is the era for humans to return to the moon, with numerous countries and space agencies conducting a series of lunar missions. Under the Artemis program, the National Aeronautics and Space Administration (NASA) is working on designing tools and developing technologies for the next lunar mission to enable long-term human exploration on the moon. China plans to accomplish a crewed lunar landing mission by 2030 and establish a human lunar base around 2045. Moreover, India, Russia, and other countries have also developed their lunar exploration plans. One of the primary concerns regarding crewed lunar exploration is the safety of astronauts during the mission. The spacecraft’s lunar-earth return is a critical phase of space missions, during which the astronauts experience high transient acceleration and sustained reentry loads, which may injure the astronauts (Erin et al., 2012). Currently, most countries use skip reentry instead of ballistic reentry with peak loads up to 16G (Graves and Harpold, 1972). There are two main types of skip reentry: one is the skip reentry of the Apollo crewed spacecraft to the moon with a peak load of about 8G, and the other is the half-skip reentry of the Chang “e manned spacecraft with a peak load of about 7–9 g (James, 1969). In both reentry modes, astronauts need to withstand acceleration loads exceeding the 3-4G load experienced during the Low Earth Orbit return. The astronauts need to endure a prolonged and highly loaded reentry environment during the spacecraft’s return. Hence, it is still one of the urgent problems to ensure astronauts” physical wellbeing during Lunar-Earth reentry.

In order to ensure the safe progress of crewed lunar missions, scholars have conducted a preliminary analysis of the damage to the body caused by sustained high acceleration loads during reentry. Khoddam-Khorasani et al. (2020) discovered that sustained high acceleration can cause mechanical low back pain due to inertial forces and even lead to spinal injuries such as vertebral fractures, spinal stenosis, intervertebral disc tears, endplate injuries, and abnormal stress in intervertebral disc, which are essential factors causing structural disorders of vertebrae and spinal instability. Additionally, studies have shown that the compression, torsion, and tensile deformation caused by high acceleration can accelerate the degenerative changes of vertebrae, potentially leading to ligament strain, annulus fibrosus rupture, and nucleus pulposus protrusion under prolonged action, affecting the astronauts’ physical health and mission execution (Zhang, 2018).

Body posture is one of the essential factors affecting the body’s capability to withstand acceleration loads. During reentry, the capsule often enters the atmosphere in a semi-ballistic skip return way, during which the seat inclination angle can affect the crew’s tolerance to acceleration. Adjusting the seat inclination angle can alter the distribution of acceleration on the spine to help the astronauts better withstand the acceleration loads (Gohmert, 2011). Ma et al. (2022) investigated the dynamic response of the Soyuz spacecraft to high acceleration with the seat angle of 20° using a dummy model, revealing the mechanism of acceleration loads transfer between different tissues when the seat was tilted. Liu et al. (2008) carried out volunteer experiments to study the acceleration response of different body segments with seat angles ranging from 10° to 50° during spacecraft landing impact, discovering a negative correlation between the peak load of Gx in the chest-back direction and the seat inclination angle. Furthermore, Pattarini et al. (2020) also suggested that an appropriate seat angle can not only increase the tolerance to acceleration loads but also significantly enhance the astronaut’s comfort during reentry into the atmosphere. Therefore, one of the urgent problems in crewed spacecraft engineering is to analyze the astronauts’ mechanical response to reentry loads under different seat inclination angles, compare the advantages and disadvantages of different schemes, and screen out the optimal scheme of seat inclination angle.

Hence, the study constructs a detailed finite element (FE) model to investigate the effects of different seat inclination angles of 0°, 10°, 20°, and 30° on the dynamic response of astronauts’ lumbar tissues under the acceleration loads of Apollo 10 and Chang’e 5T1 (CE-5T1) spacecraft returns, and assess the biomechanical differences of various seat inclination schemes in conjunction with the biological tissue damage criteria. The research findings are expected to provide a theoretical basis for designing the capsule seats in subsequent crewed lunar missions.

## 2 Materials and methods

### 2.1 FE modeling of the detailed lumbar spine

The raw CT data was collected from a China male astronaut volunteer (height: 171.4 cm, weight: 62 kg) and lumbar deformity and lesion injury were excluded. Data was collected using a 64-slice spiral CT with the volunteer in a supine position and keeping the midline of the lumbar aligned with the midline of the scan during acquisition. The collected CT data were imported into Mimics 21.0 in DICOM format for three-dimensional reconstruction of the lumbar spine using threshold segmentation and region growing. The generated STL files were then imported into Geomagic Studio software for denoising, smoothing, and other processing. Subsequently, the model underwent inspection and optimization using “Mesh Doctor” and “Accurate Surface” tools to generate NURBS surface entities. Each vertebra was then subjected to Boolean operations to construct cortical and cancellous bone, with a cortical bone thickness of 2 mm (Wang, 2018). The cortical and cancellous bone models were imported into SolidWorks 2019 in STP format. Contour curves of the intervertebral discs and facet joints were then depicted between adjacent vertebrae based on the actual physiological structure of the human body (Shirazi-adl et al., 1984). Intervertebral discs and articular cartilage were obtained using commands such as stretching and subtracting. The endplates, nucleus pulposus, and annulus fibrosus were constructed by Boolean operations and scaling, with the upper and lower surfaces of the intervertebral disc set at 0.5 mm as endplates (Zhao et al., 2018). The nucleus pulposus, an incompressible material, accounting for 48.87% of the intervertebral disc volume (Liu et al., 2006; Zhou et al., 2021).

**FIGURE 1:**
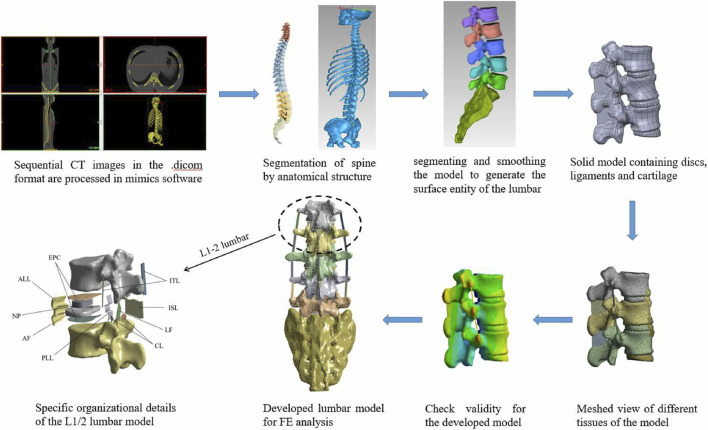
Data acquisition, image processing and detailed presentation for model development. ALL is anterior longitudinal ligament, PLL is posterior longitudinal ligament, ITL is ligamenta intertransversaria, LF is ligamentum flavum, ISL is ligamenta interspinalia, CL is Capsular ligament, IVD is intervertebral disc (containing annulus fibrosus, nucleus pulposus, and superior and inferior endplates).

Based on anatomical studies, ligaments are rugged fibrous bands that only sustain tension (Pintar et al., 1992). However, some ligaments may resist tensile forces from different directions due to their directionality. Therefore, in accordance with the different positions and fiber orientations of ligaments, corresponding cross-sectional areas were assigned in solid form near the starting and ending points to complete the construction of the ligaments, including the anterior longitudinal ligament (ALL), posterior longitudinal ligament (PLL), ligamentum flavum (LF), interspinous ligament (ISL), and intertransverse ligament (ITL). The established models included lumbar vertebrae L1-L5, sacral vertebrae (S1), and intervertebral disc (including superior and inferior endplates, nucleus pulposus, and annulus matrix). The model construction process is illustrated in Figure 1. The FE model of the lumbar spine after meshing contains 455731 elements and 751605 nodes.

According to the authentic characteristics of the lumbar spine (Iwamoto et al., 2002; Forbes, 2005; Cai et al., 2014; Wei et al., 2023), the cortical bone and cancellous bone were assigned as elastic-plastic materials, and the fibrous annulus was assigned as viscoelastic materials, with short-time shear modulus, long-time shear modulus, and elastic bulk modulus values of 32 kPa, 18 kPa, and 307 MPa, respectively. Furthermore, the intervertebral discs and small joints were constructed using linear elastic materials. The material properties of the lumbar spine are shown in Table 1. Cortical bone was set as 181 shell elements, cancellous bone as 187 solid elements, and intervertebral disc as tetrahedral elements.

**TABLE 1:** Material Parameters of each Component of Lumbar Vertebra.

Model	Tissue	E (MPa)	v	$\sigma s\;\left(\text{MPa}\right)$	$\rho \;\left(g/{\text{cm}}^{3}\right)$	$C\;\left({\text{mm}}^{2}\right)$	β	$\varepsilon \text{Pf }\left(\%\right)$	Et (GPa)	Material type
Bone	cortical bone	12000	0.3	120	1.7	—	0.1	2	1.15	elastoplasticity
	cancellous bone	40	0.3	2.2	1.1	—	0.1	3	0.01	elastoplasticity
Sacrum	cortical bone	17000	0.29	180	2	—	—	—	—	elastoplasticity
	cancellous bone	70	0.29	70	1	—	—	—	—	elastoplasticity
Intervertebral disc	annulus fibrosus	—	—	—	1.04	—	—	—	—	viscoelasticity
	nucleus pulposus	1	0.499	—	1.02	—	—	—	—	elasticity
	endplate	25	0.25	—	1.20	—	—	—	—	elasticity
Cartilage	Facet Joint	24	0.4	—	1	—	—	—	—	elasticity
Ligaments	ALL	20	0.3	—	1	63.7	—	—	—	elasticity
	PLL	20	0.3	—	1	20	—	—	—	elasticity
	ITL	58.7	0.3	—	1	3.6	—	—	—	elasticity
	LF	19.5	0.3	—	1	40	—	—	—	elasticity
	ISL	11.6	0.3	—	1	40	—	—	—	elasticity

E, elasticity modulus; v, Poisson’s ratio; σs , yield limit; ρ , density; C, cross sectional area; β , Hardening parameter; εPf , Plastic failure strain; Et, tangent modulus.

During lumbar movement, there is no separation between the vertebral bodies, intervertebral discs and upper and lower endplates, as well as annulus fibrosus and nucleus pulposus, and no relative sliding occurs between the attachment area of the ligaments and the vertebral bodies. Therefore, based on the characteristics of biological tissues, the connections are set as binding constraints (Li et al., 2018). Cortical bone and cancellous bone are connected by shared nodes. The friction between the articular surfaces is minimal and negligible due to the synovial membrane and synovial fluid in the articular capsule, and there is sliding between the facet joints and the vertebral body. Hence, a frictionless and limited sliding face-to-face contact mode was established between the facet joints and the upper vertebral body, while a binding constraint was established with the lower vertebral body (Li et al., 2022).

### 2.2 FE lumbar spine validation

This study integrated the mesh convergence method, the mechanical behaviour of cadaveric lumbar segments, and the cadaveric spinal frontal impact test to validate the effectiveness of the constructed model from the perspectives of mesh sensitivity, static deformation, and dynamic response.

#### 2.2.1 Mesh sensitivity

The FE lumbar model needs to be validated for mesh sensitivity after meshing and before being used for biomechanical analysis. According to Ayturk’s report, axial rotation was the most sensitive motion to different mesh sizes in the FE model. Therefore, models with three mesh sizes of 1 mm, 2 mm, and 3 mm were tested, respectively. Six degrees of freedom on the sacrum lower surface were constrained, and a torque of 7.5 N m was applied to the surface of L1 vertebra, analyzing the maximum stress in the lumbar tissues under different mesh schemes (Ayturk et al., 2010). The models with three mesh sizes were denoted as Mesh1, Mesh2, and Mesh3, which contained 640,302, 235,970, and 132,843 tetrahedral mesh elements, respectively. The stress differences between most tissues in each pair of mesh resolutions, as shown in Table 2, are less than 5%, indicating that the mesh divided by the FE lumbar model is effective.

**TABLE 2:** Results of the mesh sensitivity analysis.

Tissue	Stress of mesh1 (MPa)	Stress of mesh2 (MPa) (The percentage difference between Mesh1 and Mesh2)	Stress of mesh3 (MPa) (The percentage difference between Mesh1 and Mesh3)
cortical bone	58.43	59.39 (1.6%)	60.145 (2.9%)
cancellous bone	0.142	0.140 (1.4%)	0.135 (5.2%)
nucleus pulposus	0.012	0.0124 (3.3%)	0.0125 (4.2%)
PLL	0.160	0.165 (3.1%)	0.168 (5.0%)

#### 2.2.2 Static validation

Static validation of the lumbar FE model was conducted using cadaveric lumbar vertebrae motion experiments. Panjabi et al. (1994) conducted 54 groups of *in vitro* experiments to measure the lumbar spine’s range of motion (ROM) using nine fresh, frozen, intact specimens of lumbar-sacral vertebrae. The data of the flexion experiment are selected for simulation in this study.

**FIGURE 2:**
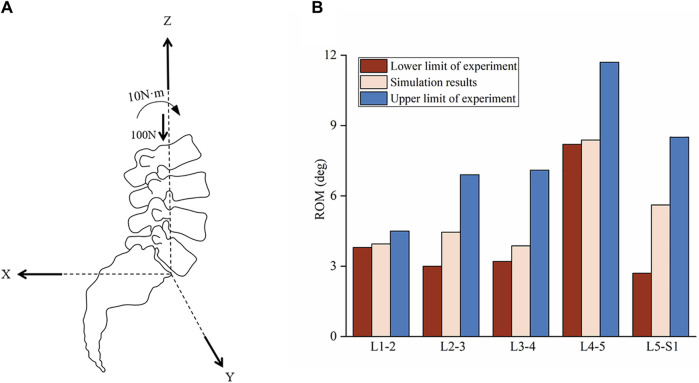
Schematic diagram of load loading and comparison of ROM in lumbar spine. **(A)** Schematic diagram of preload and torque loading, where x, y and z are the three-dimensional coordinate systems of the model motion. The origin of the coordinate system is located at the inferoposterior corner in the mid-sagittal plane of the L5 vertebra. The X-axis is pointed backwards. The Y-axis is pointed to the left, perpendicular to the sagittal plane. The Z-axis is pointed antrorsely. A preload of 100N in the negative direction of the Z-axis and a moment of 10N·m along the direction of lumbar anterior flexion were applied to the FE model with the acting point located at the L1 vertebra. **(B)** Comparison of ROM between simulation and experiments during lumbar flexion.

In the experiment, the cadaveric sacrum was fixed to the test table while the loads were applied to L1 vertebra. The remaining vertebrae were unconstrained to allow natural physiological movements of the spine to occur in response to the applied load. According to the experimental conditions, a compressive preload of 100N was applied along the Z-axis, and a 10N·m moment was applied as an external load at the centroid of the L1 vertebra, as shown in Figure 2A. The sacrum was fixed during loading, while the six degrees of freedom of other segments were freed to calculate the ROM between each lumbar segments. Figure 2B illustrates the comparison between the ROM calculated by simulation and measured in experiments, where the experimental values represent the measurements from multiple cadaver specimens. We can observe that the ROM of the model falls within the upper and lower limits of the experiment. The simulation results indicate that the model accurately simulates the motion of the lumbar spine, confirming the effectiveness of the lumbar FE model.

#### 2.2.3 Dynamic validation

The model was dynamically validated based on the axial impact cadaveric experiment conducted by Begeman et al. (1973). A total of 40 experiments were conducted using three corpse specimens, including variables of whether cushions were added and whether the deaths were normal. The data of no cushions and no blow death were selected for simulation analysis in this study.

**FIGURE 3:**
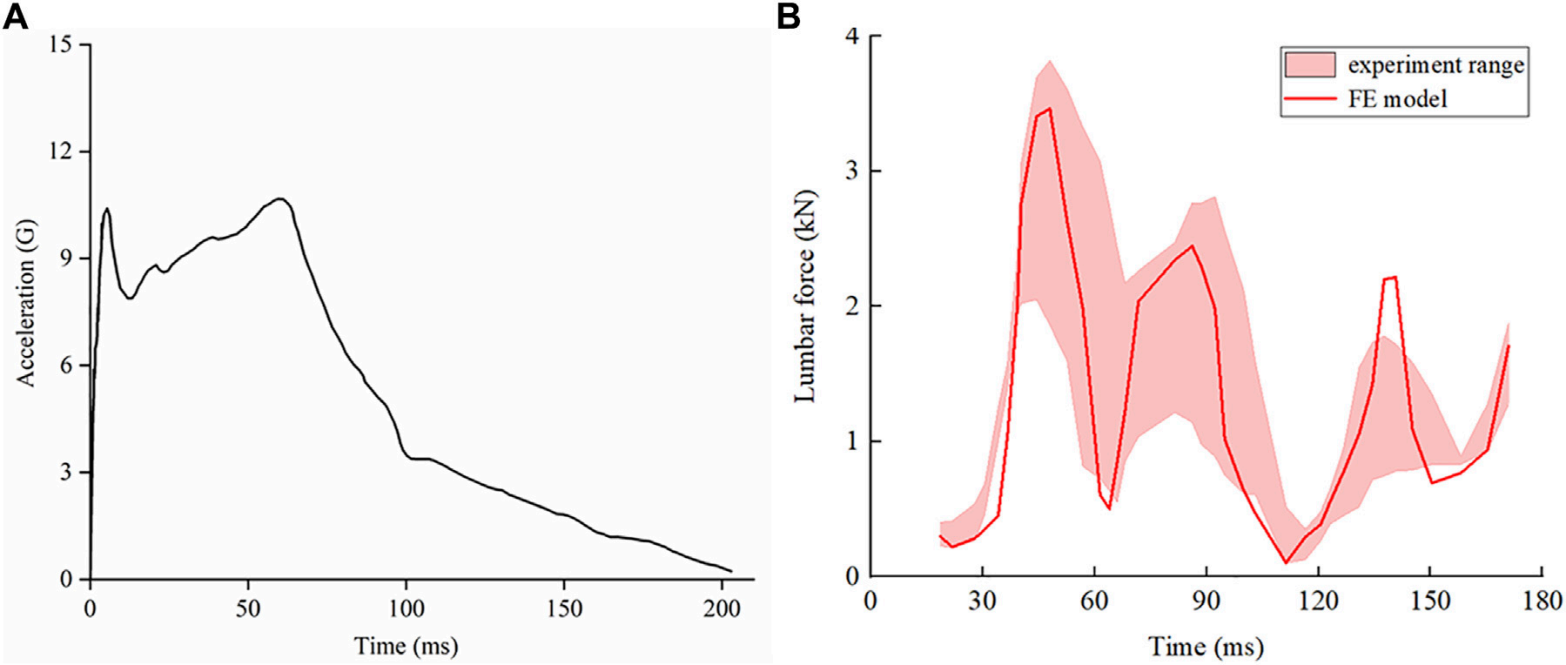
Acceleration-time curve for cadaver loading, and comparison of results predicted by simulation against cadaver experiments. **(A)** Acceleration-time curve of cadaver loading. The X-axis represents time in units of milliseconds, and the Y-axis represents the acceleration in units of gravity. **(B)** Comparison of results predicted by simulation against cadaver experiments.

Begeman et al. removed abdominal organs from the cadaver, seated the cadaver on a chair, and secured it with a belt to maintain trunk posture, preventing lateral sway of the lumbar region in response to impact. The model was fixed as a whole on a sled, with the required impact force provided by hydraulic buffers. During the simulation calculations, only the translational degrees of freedom of the sacrum in the X direction and the rotational degree of freedom in the sagittal plane were freed, with the model’s coordinate system consistent with Figure 2A. A mass point of 20 kg was coupled to the L1 vertebra to represent the trunk mass, and an acceleration load was applied to the model, as shown in Figure 3A, with the direction of chest to back, and the entire process lasting for 190 milliseconds. Based on the experimental conditions, the lumbar force data were extracted by taking the vertebrae, intervertebral discs, and ligaments as a whole. The results are shown in Figure 3B, which depicts the comparison of results predicted by simulation against cadaver experiments. Although some of the simulation data fall outside the range of the experimental threshold, the overall is consistent, and the trend of changes aligns with the experiment.

In summary, examination of the mesh convergence, static deformation, and dynamic behaviour of the FE model against the available *in silico* and *in vitro* data proves its validity. The simulation results of the lumbar FE model constructed in this study are basically consistent with the experimental data, indicating that the simulation results of this model are authentic and reliable, and the model can be utilized to investigate the biomechanical effects of the lumbar spine.

### 2.3 Model input

**FIGURE 4:**
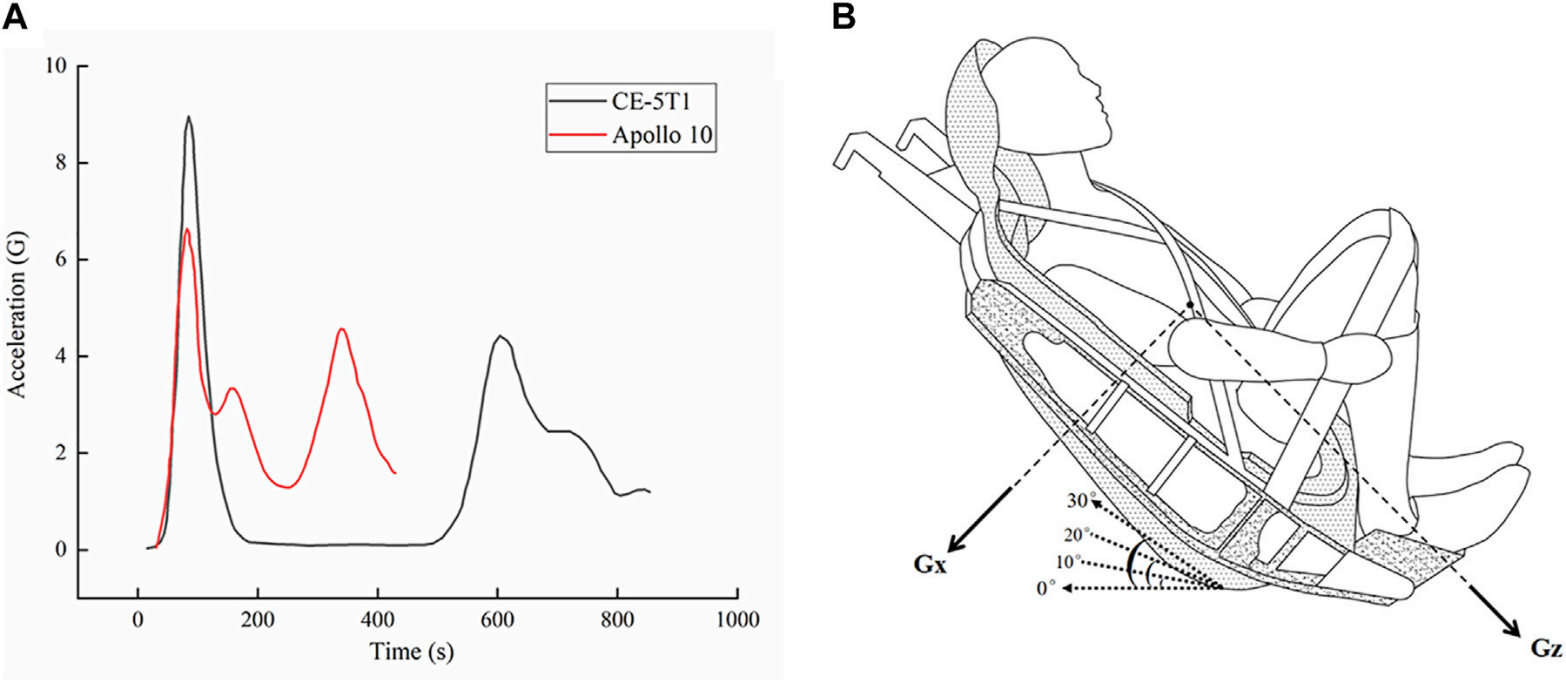
Acceleration-time curves of the Lunar-Earth reentry loads, and schematic diagram of the different seat inclination angles and load directions. **(A)** Acceleration-time curves of the Lunar-Earth reentry loads. **(B)** Schematic diagram of the different seat inclination angles and load directions, where the load of Gx is chest to back, and the load of Gz is head to foot. The seat inclination angles are 0, 10°, 20°, and 30°.

To investigate the effects of seat inclination on lumbar spine injury, the dynamic response of the lumbar spine under different seat inclination angle conditions was calculated during two types of loads, which were selected from Apollo 10 and CE-5T1 returns (Harpold and Graves, 1972; Wang, 2012). The load curve is depicted in Figure 4A. Both loads exhibited double peaks, with the first peak of 9 G and the second peak of 4 G for the CE-5T1 mission, and the first peak of 7 G and the second peak of 5 G for the Apollo 10 spacecraft. In this study, four different inclination angles were considered: 0°, 10°, 20°, and 30°, and the loads were uniformly applied to the entire lumbar spine model, with the load in the thoracodorsal direction. In the model, Gx represents the check-back direction, and Gz represents the head-foot direction. The seat inclination angles and load directions are illustrated in Figure 4B. Constrain all degrees of freedom on the sacrum lower surface, and only the X-direction degrees of freedom were released for the upper surface of the upper endplate on L1, which were used as the boundary condition in the finite element analysis. (Du et al., 2014; Park et al., 2024). There was no gravitational force involved in the entire process.

## 3 Results

### 3.1 Dynamic response of vertebrae

**FIGURE 5:**
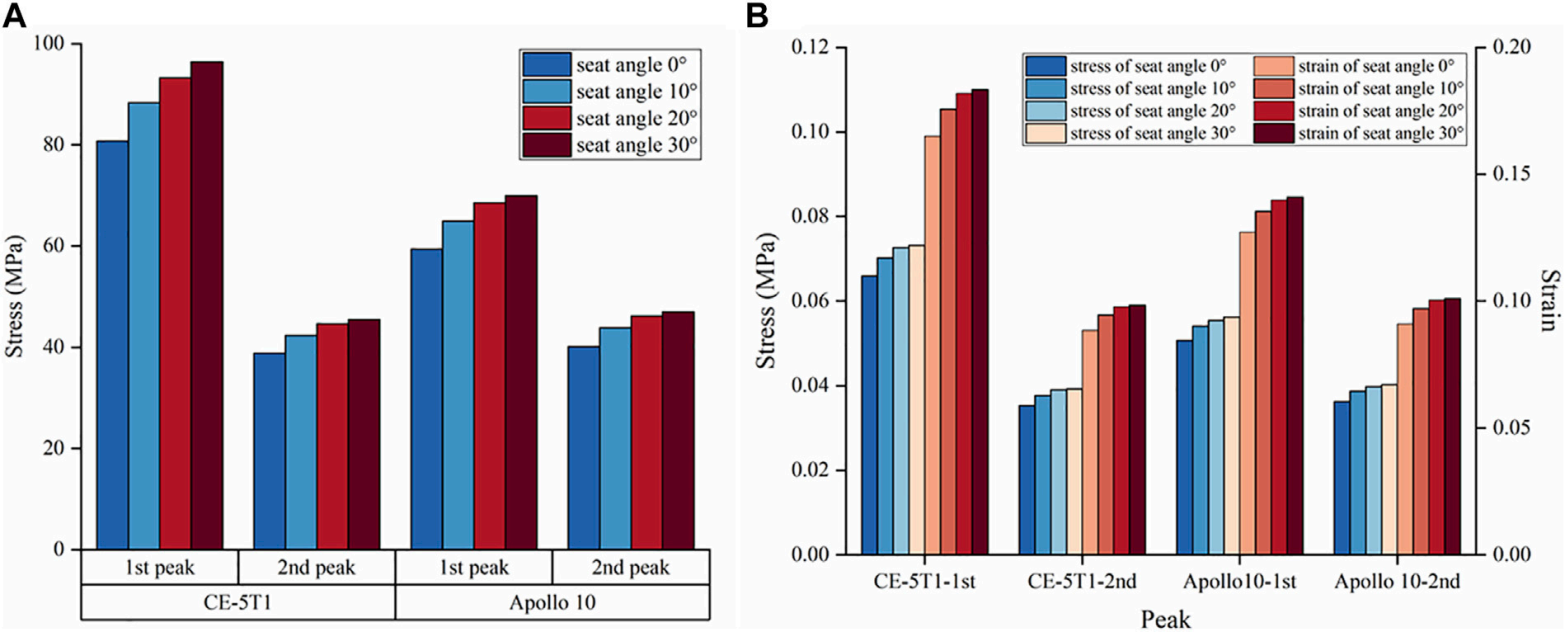
The maximum stress and strain of vertebrae under different seat inclination angles. “1st peak” represents the first peak, “2nd peak” represents the second peak. **(A)** Cortical bone stress at the load peaks of CE-5T1 and Apollo 10 under different seat inclination angles. **(B)** Stress and strain of cancellous bone at CE-5T1 and Apollo 10 load peaks under different seat inclination angles.

**FIGURE 6:**
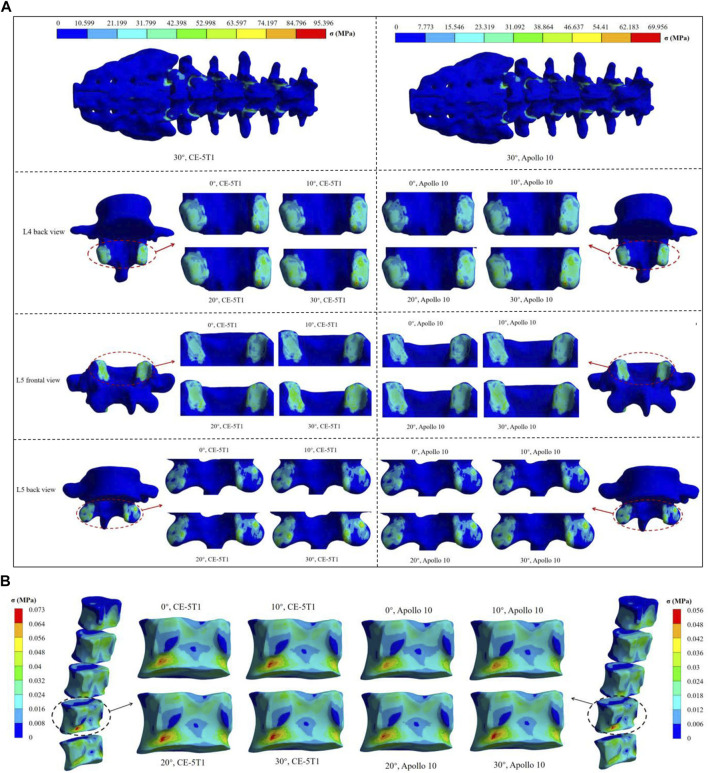
The maximum stress nephogram of vertebrae under different seat inclination angles. **(A)** stress nephogram of the superior and inferior articular processes of different vertebrae, where 0° represents a seat inclination angle of 0°, 10° represents a seat inclination angle of 10°, 20° represents a seat inclination angle of 20°, 30° represents a seat inclination angle of 30°. **(B)** stress nephogram of L4 cancellous bone.

Figure 5 presents the trends of vertebral stress and strain under different seat angles. We can observe from Figure 5A that the vertebral stress increases with the rise of the seat inclination angle, and the magnitude of increase in vertebral stress with seat inclination angle is notably higher during the first peak of CE-5T1 compared to the second peak. A similar trend of vertebral stress was observed in the Apollo 10 mission, where higher loads resulted in a more pronounced variation in vertebral stress with seat inclination angle. According to the study by Carter and Hayes (1976), the formula for calculating human bone strength is defined as

$$S=68{\left(d\varepsilon /dT\right)}^{0.06}\rho_{\alpha }^{2},$$ (1)

with the cortical bone strength limit given as 227 MPa, where S is the bone mineral density, $\rho_{\alpha }$ is the bone density, dε/dT is the strain rate. The vertebral stress values under different seat angles are below the strength limit of human cortical bone, indicating no risk of vertebral injury. As shown in Figure 6A, the superior and inferior articular processes of the vertebrae experience more significant stress, particularly in the regions of L4-L5 and L5-S1. This is attributed to the shorter height of vertebrae in the lower lumbar and the small distance between the superior and inferior articular processes. Repeated loading and unloading of the loads during reentry can easily cause fatigue damage to the vertebrae, resulting in limited spinal function (Orwoll et al., 2013).

According to the relevant mechanical experiments (Morgan et al., 2003; Sevilla et al., 2007), the ultimate strength of cancellous bone ranged from 2 to 12 MPa, and the failure strain was 1.3% (Zheng et al., 2018). Cancellous bone stress and strain increase with the rise of the seat inclination angle, reaching maximum stresses of 0.073 MPa and 0.056 MPa during the loads of CE-5T1 and Apollo 10 spacecraft, respectively, lower than the ultimate strength of cancellous bone. The maximum strain values are 0.18% and 0.14%, respectively, below the failure strain of cancellous bone (Figure 5B). Neither load directly affects the interior structure of the vertebral body. However, as illustrated in Figure 6B, the stress on the posterior lower edge of the L4 cancellous bone is more pronounced, increasing with the increase of the seat inclination angle. Prolonged exposure to loads can lead to stress concentration, cumulative micro-damage, degradation of mechanical properties, and degeneration of cancellous bone structure. Therefore, the protection of the L4 cancellous bone should be enhanced.

### 3.2 Dynamic response of annulus fibrosus and nucleus pulposus

**FIGURE 7:**
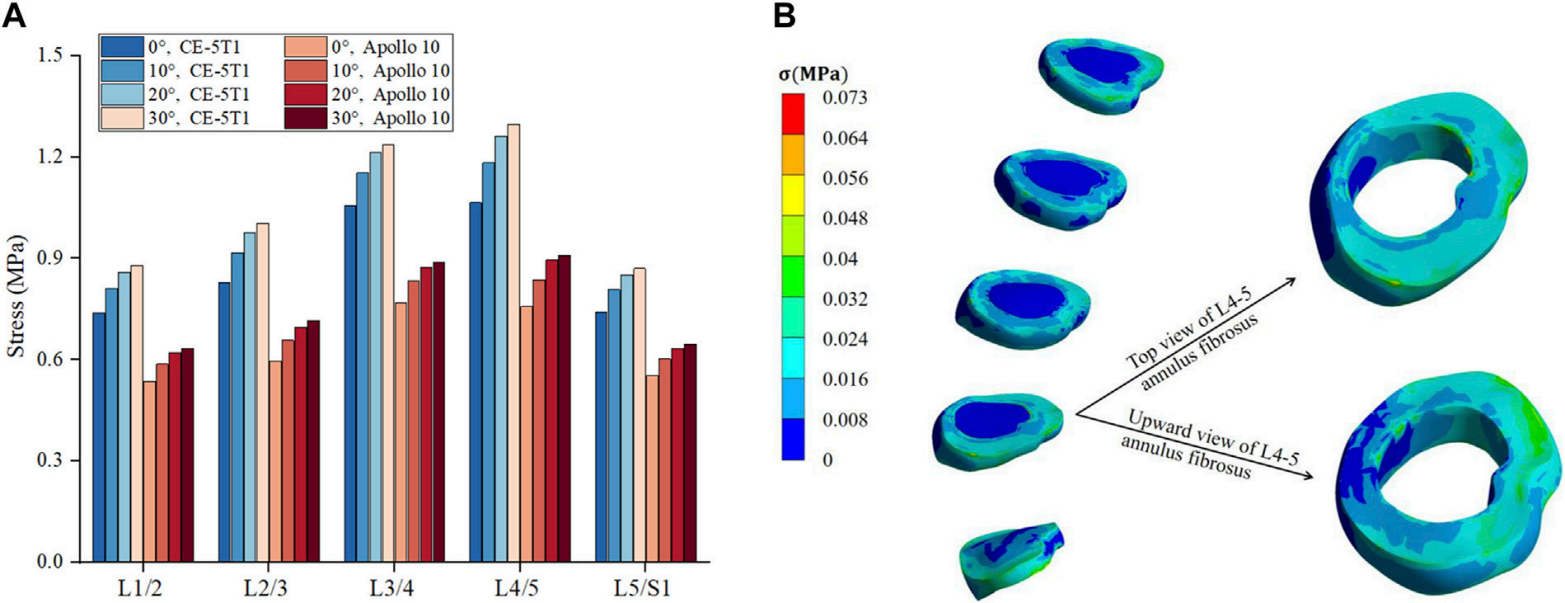
Comparison of the maximum stress on fibrous annulus and nucleus pulposus under different seat inclination angles. **(A)** Stress variation of fibrous annulus at CE-5T1 and Apollo 10 reentry loads under different seat inclination angles, where 0° represents a seat inclination angle of 0°, 10° represents a seat inclination angle of 10°, 20° represents a seat inclination angle of 20°, 30° represents a seat inclination angle of 30°. **(B)** Stress nephogram of the fibrous annulus and nucleus pulposus, taking the seat inclination angle of 30° under the reentry load of CE-5T1 as an example.

Figure 7A reveals that the stress in the intervertebral disc is primarily concentrated on the annulus fibrosus. During the Apollo 10 and CE-5T1 missions, the stress on each segment of the annulus fibrosus increases with the rise of the seat inclination angle, and the growth rates are 9.5% and 10% at seat inclination angles of 0° and 10°, respectively, while at seat inclination angles of 20° and 30°, they both are 2.3%. Moreover, within the seat inclination angles from 0° to 30°, the annulus fibrosus stress values in CE-5T1 are higher than those in Apollo 10.

Under the same seat inclination angle, the stress values of the annulus fibrosus exhibit an increasing trend from the top to the bottom. L4 and L5 vertebrae are located in the bulge of the physiological curvature of the human spine, with more considerable intervertebral mobility, making the L4/5 intervertebral disc more susceptible to injury. Skaggs et al. (1994) reported that the maximum stress human annulus fibrosus could withstand ranged from 24 to 44.8 MPa. The maximum stress values on the annulus fibrosus under CE-5T1 and Apollo 10 are 1.30 MPa and 0.91 MPa, respectively, far below the stress threshold the annulus fibrosus can withstand.

The stress in the nucleus pulposus follows a similar trend to the annulus fibrosus, with an initial increase followed by a decrease, but the stress peak differs significantly from those of the annulus fibrosus. Under both reentry loads, the stress concentration in the nucleus pulposus mainly occurs in the posterior region. Figure 7B illustrates that the stress on the L4/5 annulus fibrosus is mainly distributed in the rear, and the structure of the posterolateral annulus fibrosus is weaker. Prolonged loading and unloading of loads can lead to the protrusion of the nucleus pulposus (Marchand and Ahmed, 1989).

### 3.3 Dynamic response of ligaments

**FIGURE 8:**
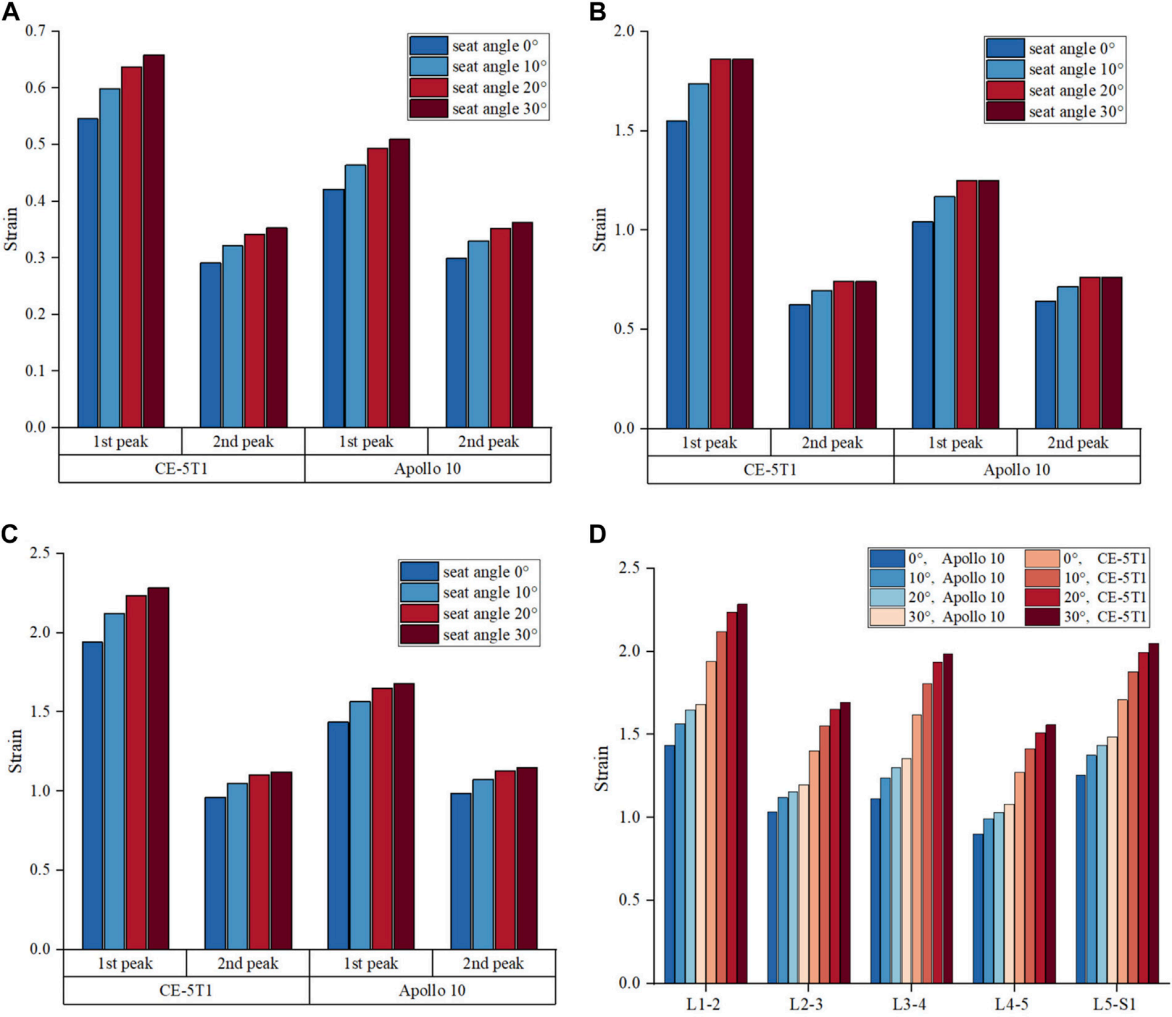
The maximum strain of ligament at the load peaks of CE-5T1 and Apollo 10 under different seat inclination angles. **(A)** Strain of the LF. **(B)** Strain of the ALL. **(C)** Strain of the PLL. **(D)** Strain of the PLL at different segments of vertebrae, where 0° represents a seat inclination angle of 0°, 10° represents a seat inclination angle of 10°, 20° represents a seat inclination angle of 20°, 30° represents a seat inclination angle of 30°. L1-2 represents the PLL located between the L1 and L2 vertebrae, L2-3 represents the PLL located between the L2 and L3 vertebrae, L3-4 represents the PLL located between the L3 and L4 vertebrae, L4-5 represents the PLL located between the L4 and L5 vertebrae, L5-S1 represents the PLL located between the L4 vertebrae and the sacrum.

Figure 8 illustrates the trend of ligament strain under different seat inclination angles. Under the same acceleration, the maximum strain of the ALL, PLL, and LF is higher during the first peak compared to the second peak. Additionally, the strain value of the LF, which has higher elasticity and strength (Dumas et al., 1987), shows a relatively smaller variation with seat inclination angle. Under the load of Apollo 10, the strain of LF is positively correlated with the seat inclination angles from 0° to 20°, and the strain value remains almost unchanged at seat inclination angles from 20° to 30° (Figure 8A). The strain values of the ALL and PLL increase as the seat inclination angles range from 0° to 30°. Under the load of CE-5T1, there is no significant difference in the strain values of the LF at seat inclination angles of 20° and 30°, while the strain value of the ALL reaches a minimum at the seat inclination angle of 10°. The minimum strain values of the ALL under the two peaks are 1.55 and 0.62 respectively, occurring at the seat inclination angle of 10°, while the maximum strain values are 1.92 and 0.76 respectively, occurring at the seat inclination of 30° (Figure 8B). Under the two peak conditions, the stress differences caused by seat inclination angle are 24% and 20%, respectively. Under the same load, the higher the seat inclination angle, the more pronounced the ligament strain.

Figure 8D indicates that within the seat inclination angles from 0° to 30°, the strain values of the PLL in different segments increase as the seat inclination angle rises under the two reentry loads, but the increasing rates decrease. Under both reentry loads, the strain values of the PLL in different segments increase by approximately 9%–12% when the seat inclination angles range from 0° to 10°, approximately 4%–7% when the seat inclination angles range from 10° to 20°, and approximately 2%–4% when the seat inclination angles range from 20° to 30°. The PLL exhibits larger strain values at the L1-2, L3-4, and L5-S1 segments. Prolonged high loads can induce progressive stress injuries to the ligaments, disrupt the load distribution in the lumbar spine, increase stress on the remaining ligaments, and affect spinal stability (Li et al., 2023). For the load of CE-5T1, the strain on the PLL continues to increase when the seat inclination angle increases to 30° (Figure 8C). Therefore, it is advisable to enhance the protection of the PLL at the L1-2 segment at high seat inclination.

### 3.4 Assessment of lumbar tissue injury

Studies have shown that the Dynamic Response Index (DRI) can be used to calculate the maximum compression of the human spine under z-direction loads, characterizing the tolerance limit of the human body to loads (Stech and Payne, 1969). The calculation formula is shown in (2), where $\delta_{\max}$ is the maximum deformation of the spine. Since changes in seat inclination angles can affect the distribution of loads in the spine, it is necessary to consider the physiological tolerance of loads in three directions. Hence, the Multi-Axis Dynamic Response Criterion (MDRC) proposed by the Armstrong Laboratory of the United States was introduced to analyze spinal injury under reentry load conditions. This Criterion assumes the human-seat system as a damping spring model, and then characterize the effects of acceleration load on the human body (McDonald, 1998). MDRC is currently the most advanced internationally recognized standard used to assess the tolerance of astronauts to acceleration. The specific formula is shown in (3).

$$\text{DRI}=285.26\;\delta_{\max}$$ (2)

$$\text{MDRC}=\sqrt{{\left(\frac{{DRI}_{X}}{{DRI}_{XL}}\right)}^{2}+{\left(\frac{{DRI}_{Y}}{{DRI}_{YL}}\right)}^{2}+{\left(\frac{{DRI}_{Z}}{{DRI}_{ZL}}\right)}^{2}}$$ (3)

In the formula, ${DRI}_{X}$ , ${DRI}_{Y}$ , ${DRI}_{Z}$ are the components of the dynamic response experienced by the crews in the three axis directions. ${DRI}_{XL}$ , ${DRI}_{YL}$ , ${DRI}_{ZL}$ are the corresponding tolerance limits (Stewart et al., 2004), where the forward and backward acceleration limits on the X-axis are 40 and 35 respectively, the upward and downward acceleration limits on the Y-axis are 18 and 16.5 respectively, and the acceleration limits on the Z-axis is 17. The acceleration tolerance of the human body can be reflected through MDRC. When MDRC >1, it is considered to have exceeded the body’s tolerance standard, indicating a higher probability of body injury.

**FIGURE 9:**
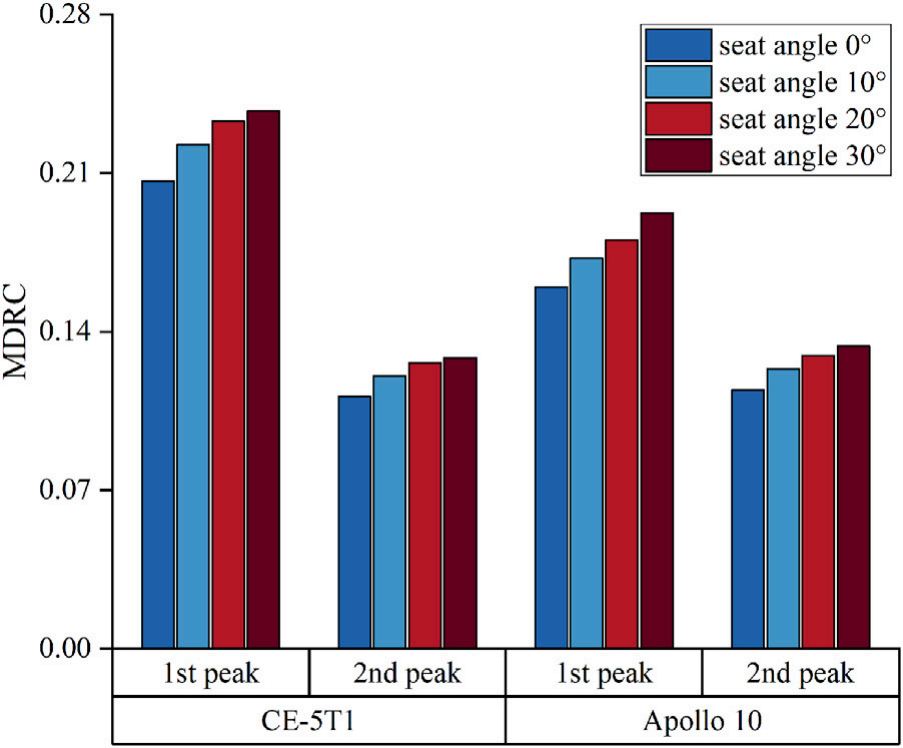
MDRC of lumbar spine at the load peaks of CE-5T1 and Apollo 10 under different seat inclination angles.

Figure 9 illustrates the results of the MDRC at the load peaks of CE-5T1 and Apollo 10 under different seat inclination angles. At the first peak, the growth rate of MDRC for the CE-5T1 spacecraft is notably higher than that for the Apollo 10 spacecraft. At the second peak, the growth rate of MDRC for Apollo 10 is slightly higher than that for CE-5T1, but the overall growth rates are similar.

The MDRC of the lumbar spine under the four seat inclination angles is lower than the injury threshold of 1, indicating that the loads will not cause injury to the human spine within the seat inclination angle of 30°during both the reentry of CE-5T1 and Apollo 10. However, to ensure the safety of astronauts and maximize the protection effectiveness of the protection system, it is advisable to minimize the MDRC.

## 4 Discussion

With the continuous development of crewed lunar exploration, studying of load injuries and protective measures for astronauts during lunar missions has attracted widespread attention among scholars. In this study, based on the data obtained from the lumbar FE model simulation calculations, quantitative analyses were conducted on the astronauts’ dynamic responses and injury risks under different seat inclination angles during Lunar-Earth reentry return in conjunction with the biological tissue injury criteria. The research findings indicate that, as the seat inclination angle increases, the stress values on the vertebrae of both the CE-5T1 and Apollo 10 spacecraft significantly increase, with a more pronounced overall damage risk observed in the CE-5T1 spacecraft. However, under the four seat inclination conditions, the tissue stress of astronauts is within the threshold value and will not directly cause injury.

The analysis of the simulation results reveals that the variation trend of vertebral stress near the peak of Apollo 10 is similar to that of CE-5T1 under the four seat inclination angles, increasing gradually with the rise of the seat inclination angle, and the increasing rate gradually decreases. The growth rates of vertebral stress at the seat inclination angles of 0° and 10° are 9% and approximately 5% at the seat inclination angles of 10° and 20°, which indicate that when the seat inclination angle of the return capsule initially increases within 10°, the stress on astronaut tissues increases due to the Z-component effect (Honkanen et al., 2018). When the seat inclination angle rises to 20°, the increasing rate of tissue stress slows down. This observation aligns with Sonenblum’s (2009) findings, indicating significant vertebral stress variations when the seat reclined initially, with no significant response of human tissues when the seat was reclined at 15° and 20°. On the other hand, during the first peak, the growth rates of vertebral stress at seat inclination angles from 20° to 30° are 3.4% in the CE-5T1 spacecraft, compared to only 2.1% in the Apollo 10. During the second peak, the growth rates of vertebral stress for CE-5T1 and Apollo 10 tend to remain consistent at seat inclination angles ranging from 20° to 30°. This is because the first peak of CE-5T1 is nearly 9 g, which is 2.3 g higher than the first peak of Apollo 10. When the peak is high, the human tissue is significantly affected by the seat inclination angles that exceed 20°. The component effects of high acceleration load in the Gz direction on the human body will increase with the rise of the seat inclination angle, making it easier to reach the tolerance limit of astronauts (Fan and Liu, 2010). In addition, the stress and strain on the L4 cancellous bone also increase with the seat inclination angle. This is because the L4 vertebra is located at the vertex of the lordotic position of the spine’s physiological curvatures, and the L5 vertebra is relatively fixed, resulting in a wide range of motion of L4 and making it susceptible to cause stress concentration (Kalichman et al., 2009). Compared to Apollo 10, the higher peak of CE-5T1 results in a more pronounced response of astronauts to the seat inclination. Considering the astronauts’ physical decline after long-duration space missions, it is recommended that the first peak of CE-5T1 reentry should be designed not to exceed the first peak of Apollo 10.

The intervertebral disc, an essential component of the lumbar spine, serves the functions of supporting and connecting the vertebrae, as well as cushioning external impacts (Delgado-López et al., 2017). Common injuries to the intervertebral disc often occur in the annulus fibrosus. As the seat inclination angle varies from 0° to 30°, the stress on the annulus fibrosus under CE-5T1 and Apollo 10 increases, with the maximum stress located at the rear side of the annulus fibrosus. The repeated loading and unloading of loads in regular training can lead to stress concentration and thus injuries. The literature also indicated (Qasim et al., 2012) that intervertebral disc injuries under cyclic loading mainly originate from the posterior side of the annulus fibrosus, and the injuries tend to worsen with an increasing number of loading cycles. Prolonged and repeated loads tend to induce stress concentration, resulting in fatigue damage to the annulus fibrosus and premature degeneration of the intervertebral disc (Bailey et al., 2022; Kelekis et al., 2022). The stress characteristics of the annulus fibrosus with changes in seat inclination angle are similar to the stress patterns observed in the vertebral bodies, with stress values directly proportional to the seat inclination angle. Under both acceleration conditions, the stress concentration occurs in the annulus fibrosus and nucleus pulposus between the L4 and L5 vertebrae, accompanied by significant strain values. Long-term repeated damage to the L4-5 annulus fibrosus can lead to protrusion of material inside the disc and compression of adjacent nerves, resulting in lower back pain or restricted mobility, affecting astronauts’ task performance and overall safety (Pollock et al., 2021).

Ligaments, like the vertebrae, intervertebral discs, and facet joints, are essential in maintaining the spine’s stability. Comparing the mechanical response of ALL, PLL, and LF under different seat inclination angles, we discovered that the PLL had the highest strain values under the load of CE-5T1 and exhibited the most pronounced response to the change in seat inclination angle. The high sensitivity to changes in seat inclination is attributed to the characteristics of the PLL in supporting spinal mobility, stability, and flexibility (Yoshikane et al., 2020). Consequently, the effects of high acceleration will first appear in the PLL. These differences resulting from seat inclination angles can be explained by the Gz acceleration response, where within a specific range of angles, as the seat inclination angle increases, the decomposition of acceleration in the Z component increases. Relevant studies (Pollock et al., 2021) on human acceleration tolerance indicate that the human body exhibits higher tolerance to Gx acceleration than Gz acceleration. Prolonged exposure to high Gz environment can lead to ligament stretching and laxity, which can eventually result in ligament injury, spinal instability, and other diseases. Compared with the seat inclination angle of 20°, when the seat inclination angle increases to 30°, there is a slight increase in strain values in different segments of the PLL during the Apollo 10 mission, but no significant difference occurs. For the Apollo 10 spacecraft, seat inclination angles within 30° do not directly harm the astronauts, but as the seat inclination angle increases, it is still necessary to enhance the astronauts’ spinal protective design. Similar to the vertebrae response, the strain values at different segments of the PLL under the second peak of CE-5T1 are lower than those under the second peak of Apollo 10. This difference can be attributed to the prolonged exposure to the near-zero gravity Kepler segment of the CE-5T1, which can effectively buffer and protect spinal tissues from injury. Therefore, adding the Kepler phase during the return of Apollo 10 is advisable to protect astronauts from load-induced injury.

Burns (1975) discovered that within a specific range of angles, the human body’s tolerance decreases with the increased seat inclination angle. Similarly, the simulation results indicate that under the same acceleration conditions, the stress values on the vertebrae and intervertebral discs are notably higher at the seat inclination angle of 30° compared to 0°, particularly in the region from L3 to L5 vertebrae. Moreover, under the same seat angle condition, the mechanical responses of cancellous bones and ligaments during the first peak of the CE-5T1 mission are significantly greater than that of the Apollo 10 mission, suggesting that the increased Z-component and high accelerations resulting from high seat angles are likely to cause stress concentration and acute injuries. Stress-induced injuries due to high loads have also been observed in animal experiments. Clarke et al. (1972) demonstrated that baboons experience compressive vertebral fractures even at high deceleration levels (6.5 g–20 g), with fatal injuries primarily dependent on the magnitude of the peak rather than deceleration time. Hence, it is recommended that the inclination angle of the astronaut seat during the return of CE-5T1 be set below 30°. When the seat inclination angle exceeds 30°, the spacesuit design should be optimized to enhance protection for the L4 vertebra and adjacent structures, ensuring maximum safety for the astronauts.

The limitation of this study is that the lumbar spine finite element model did not use sitting alignment data. The lumbar spine alignment is different in the supine and seated postures, which may result in differences in the data calculated by the lumbar spine model in the supine position and the seated position. Therefore, ongoing research will gradually improve the lumbar spine model to enhance the accuracy and reliability of the study findings.

To comprehensively assess the biomechanical effects of different seat inclinations on astronauts during reentry and return, further refinement of the model’s tissue representations and a more detailed categorization of seat inclination angles should be considered to make the biomechanical analysis results more accurate.

## 5 Conclusion

In this study, we proposed four schemes of seat inclination angle to analyze the dynamic response of lumbar tissue under reentry loads of Apollo 10 and CE-5T1 and quantitatively evaluated the lumbar tissue injury under different seat inclination angles in combination with the biological tissue injury criteria. The study results provide a numerical basis for analyzing the potential injury mechanisms associated with seat inclination angle during the Lunar-Earth reentry return, offering theoretical support for designing the seat angles, developing protective equipment, and improving the seatback restraint system to minimize the risk of injury to astronauts.

## Data Availability

The original contributions presented in the study are included in the article/Supplementary Material, further inquiries can be directed to the corresponding authors.
